# Brain Molecular Connectivity in Neurodegenerative Diseases: Recent Advances and New Perspectives Using Positron Emission Tomography

**DOI:** 10.3389/fnins.2019.00617

**Published:** 2019-06-14

**Authors:** Arianna Sala, Daniela Perani

**Affiliations:** ^1^Faculty of Medicine and Surgery, Vita-Salute San Raffaele University, Milan, Italy; ^2^Division of Neuroscience, Faculty of Psychology, San Raffaele Scientific Institute (IRCCS), Milan, Italy; ^3^Faculty of Psychology, Vita-Salute San Raffaele University, Milan, Italy; ^4^Nuclear Medicine Unit, Faculty of Psychology, San Raffaele Hospital (IRCCS), Milan, Italy

**Keywords:** amyloid PET, brain networks, connectivity, FDG–PET, multivariate analysis, neurodegenerative diseases, neurotransmission, tau PET

## Abstract

Positron emission tomography (PET) represents a unique molecular tool to get *in vivo* access to a wide spectrum of biological and neuropathological processes, of crucial relevance for neurodegenerative conditions. Although most PET findings are based on massive univariate approaches, in the last decade the increasing interest in multivariate methods has paved the way to the assessment of unexplored cerebral features, spanning from resting state brain networks to whole-brain connectome properties. Currently, the combination of molecular neuroimaging techniques with multivariate connectivity methods represents one of the most powerful, yet still emerging, approach to achieve novel insights into the pathophysiology of neurodegenerative diseases. In this review, we will summarize the available evidence in the field of PET molecular connectivity, with the aim to provide an overview of how these studies may increase the understanding of the pathogenesis of neurodegenerative diseases, over and above “traditional” structural/functional connectivity studies. Considering the available evidence, a major focus will be represented by molecular connectivity studies using [18F]FDG–PET, today applied in the major neuropathological spectra, from amyloidopathies and tauopathies to synucleinopathies and beyond. Pioneering studies using PET tracers targeting brain neuropathology and neurotransmission systems for connectivity studies will be discussed, their strengths and limitations highlighted with reference to both applied methodology and results interpretation. The most common methods for molecular connectivity assessment will be reviewed, with particular emphasis on the available strategies to investigate molecular connectivity at the single-subject level, of potential relevance for not only research but also diagnostic purposes. Finally, we will highlight possible future perspectives in the field, with reference in particular to newly available PET tracers, which will expand the application of molecular connectivity to new, exciting, unforeseen possibilities.

## Introduction

During the last decades, positron emission tomography (PET) has established itself as a relevant tool, in providing *in vivo* biomarkers for neurodegenerative diseases associated with cognitive decline and dementia, and playing a leading role in the diagnostic work-up of these conditions (Albert et al., 2011; Gorno-Tempini et al., 2011; McKhann et al., 2011; Rascovsky et al., 2011; Sperling et al., 2011; Armstrong et al., 2013; Dubois et al., 2014; McKeith et al., 2017). PET represents a unique tool to *in vivo* measure different molecular processes that are key to the pathophysiology of neurodegenerative conditions (cf. Iaccarino et al., 2017b). Together with well-established tracers, such as [18F]FDG, measuring cellular glucose metabolism, new tracers have and are being developed, providing access to a widespread set of biological and pathological processes, from neurotransmission to amyloid and tau pathology.

Recently, the field of neurodegenerative diseases has witnessed a paradigmatic shift, with the research focus shifting from evaluating the effect of underlying pathology on *local* neuronal function to assessing the long-distance effects of brain pathology on interconnected neural systems (Fornito and Bullmore, 2015). Pathophysiological models of neurodegeneration now take into account brain inter-regional anatomical and functional networks, considered as relevant targets of pathology, on the one hand (Palop et al., 2006), and as key players in pathology spreading, on the other hand (Seeley, 2017).

The knowledge on functional and structural brain networks and connectivity is increased rapidly, with a plethora of studies focusing on magnetic resonance imaging (MRI), as a widely available and cost-effective *in vivo* tool (see for review Fornito and Bullmore, 2015; Fornito et al., 2015).

Information on molecular brain networks and connectivity, as assessed by PET, is still scarce, with the few studies mostly focusing on [18F]FDG–PET metabolic connectivity. Here we review the most recent advances in this emerging field. Following a brief introduction on the available PET tracers and on the theoretical and methodological framework of brain connectivity, we will review available molecular connectivity studies using PET, particularly in combination with [18F]FDG tracer, as a functional measure of brain metabolism. Pioneering studies assessing molecular connectivity with tracers for neurotransmission and brain pathology will also be discussed. Finally, methodological advances and future directions in the field will be reviewed.

## PET: Relevant Tracers for Neurodegenerative Diseases

Positron emission tomography studies of neurodegenerative diseases have greatly contributed to the research in clinical neuroscience (Jagust, 2018), by providing access to a series of molecular measures impossible to obtain *in vivo* with other neuroimaging techniques (Iaccarino et al., 2017b). PET is increasingly showing its potential in supporting clinical diagnosis of neurodegenerative conditions, also in the early, if not preclinical, disease phases, by allowing the detection of subtle pathological and functional neural changes even before clinical symptoms become manifest (Albert et al., 2011; Sperling et al., 2011).

Traditionally, the focus of brain PET studies has been on *brain metabolism*, as accurately measured by [18F]FDG–PET. It is well-established that [18F]FDG–PET signal, reflecting both oxidative metabolism in neurons and aerobic glycolysis in astrocytes, is strictly coupled to synaptic function (Stoessl, 2017) and dysfunction. Since synaptic dysfunction can arise from several neuropathological events, among which altered intracellular signaling cascades and mitochondria bioenergetics, impaired neurotransmitter release, accumulation of neurotoxic protein species, and long-distance disconnections (Perani, 2014), [18F]FDG–PET can be considered as a “funnel” biomarker, able to capture all the different pathological events that produce a perturbation in glucose metabolism. Decades of research have shown that specific patterns of hypometabolism can be consistently detected in the major neurodegenerative conditions, from Alzheimer’s disease to dementia with Lewy bodies to the different syndromes of the frontotemporal dementia spectrum (cf. Perani et al., 2014; Cerami et al., 2015, 2016; Caminiti et al., 2019).

Positron emission tomography can also be used to measure receptor density (both at pre- and post-synaptic level) and transporter binding in *neurotransmission systems*. PET tracers have been developed for measuring the integrity of several brain neurotransmission systems, from the aminenergic to the μ-opioid systems. Although the major application of tracers for neurotransmission is in the field of psychiatric disorders, some of these tracers have been used to measure neurochemical alterations in neurodegenerative diseases. Among the most used tracers, [11C]MP4A – and analogous tracers for cholinergic presynaptic function – have shown reduced acetylcholinesterase activity in the cortex, hippocampus, and amygdala in Alzheimer’s disease (Herholz et al., 2004; Marcone et al., 2012), and even more severe reductions in dementia with Lewy bodies and Parkinson’s disease with dementia (Klein et al., 2010). In parallel, PET studies targeting the dopaminergic system have allowed to consistently show, *in vivo*, reduced dopaminergic transporter activity – a transmembrane protein regulating extracellular levels of dopamine – in Parkinson’s disease and atypical Parkinsonian conditions (Varrone and Halldin, 2010; Caminiti et al., 2017b). Still, the use of these tracers, usually carbon-labeled and thus requiring the presence of a cyclotron, on-site, is limited to research studies, with more restricted applications in daily clinical practice.

More recently, efforts in tracer development have focused on targeting brain aggregates of *pathological proteins*, with successful validation of tracers for amyloid and (partially) tau pathology, representing the key pathological aggregates of Alzheimer’s disease (Jack et al., 2018). The binding properties of currently available *amyloid* tracers have been well-characterized, with tracers binding selectively and with high affinity to the β-sheet structure of fibrillary amyloid plaques, with low affinity to diffuse plaques and showing no affinity for other amyloid isoforms, such as protofibrils or oligomers (Fodero-Tavoletti et al., 2012; Ni et al., 2013; Sabri et al., 2015). The availability of these *in vivo* markers for amyloid pathology has greatly improved the design of clinical trials for Alzheimer’s disease, advancing strategies for patients’ selection and allowing *in vivo* evaluation of target engagement (Vandenberghe et al., 2013). As for *tau* tracers, available data derive mainly from first-generation tau tracers, binding with high affinity to tau neurofibrillary tangles (Fodero-Tavoletti et al., 2011; Xia C.F. et al., 2013; Hashimoto et al., 2014). Preliminary studies have shown that tau PET imaging could be a valuable tool for the *in vivo* staging of Alzheimer’s disease pathology progression (Schöll et al., 2016; Schwarz et al., 2016). Still, major areas of concern remain, regarding the type of tau pathology being targeted, i.e., 3-repeat vs. 4-repeat tau isoforms, and presence of non-specific binding in the striatum and choroid plexus, and the off-target binding to neuromelanin and monoamine oxidase (MAO-A/B) (Saint-Aubert et al., 2017; Lemoine et al., 2018).

## The Brain as a Network

Although most imaging findings are based on massive univariate approaches, the increasing interest in multivariate methods has paved the way to the assessment of unexplored cerebral features, spanning from resting state brain networks to whole-brain connectome properties. The advantage of using multivariate methods is that they allow to assess variations in the *relationship* between brain regions, over and above *local* regional changes, measureable with univariate methods (Clark and Stoessl, 1986). The great majority of multivariate findings derive from structural and functional MRI (fMRI) studies, respectively, providing information on brain axonal pathways, and on the correlation of blood-oxygen-level-dependent (BOLD)-signal time course across brain regions. Still, the first seminal studies assessing covariations in brain function were performed, already in the 1980s, using brain metabolic data derived from [18F]FDG–PET (Horwitz et al., 1984, 1987). From the 1990s, the popularity of multivariate approaches steeply increased, following the development of fMRI, and the observation that spontaneous activity in the primary motor cortex correlates with the activity of a widespread, spatially distributed, network of brain regions (Biswal et al., 1995). Later, based on [18F]FDG–PET evidence of coherent metabolic decreases during cognitive tasks vs. resting condition, it was hypothesized that different sets of brain regions organize into different brain networks (Raichle et al., 2001). Subsequent fMRI studies confirmed that other large-scale networks, whose regions show coherent patterns of dynamic activity, exist in the “resting brain” (Greicius et al., 2003) and that brain spontaneous activity can essentially be decomposed in a series of internally coherent large-scale functional brain networks (Beckmann et al., 2005). From the 2000s, building on this evidence, and borrowing methodological tools from the field of graph theory, a new theoretical framework was proposed, under the name of “connectomics” (Sporns et al., 2005). This framework, also known as the new “systems biology of the brain,” uses graph theory indices to investigate the properties of the brain functional and structural architecture, on the assumption that a comprehensive characterization of the brain as a network is necessary to understand brain function (and dysfunction) (Sporns et al., 2005). In recent years, these methodological advances have been further extended to PET data. An excellent review of the analysis methods cited in this paragraph, and their adaptation to PET imaging data, is also available (Yakushev et al., 2017).

In the study of neurodegenerative diseases, the relevance of modeling the brain as a system of interconnected regions spans from two foundational hypotheses, one conceptualizing brain networks as *passive targets* of brain pathology (Palop et al., 2006) and the other as *active players* in the spreading of pathology (Seeley, 2017).

In the first “*passive*” conceptualization, brain networks are deemed relevant targets of brain pathology, dynamically altered by plasticity mechanisms that arise from pathological processes (Palop et al., 2006). It is assumed that pathological processes not only alter activity of isolated regions, but also produce distributed effects on brain networks, by prompting a reorganization of regional interconnections through induction of dedifferentiation and compensation processes (Fornito et al., 2015). A decade of evidence indeed suggests that the effects of molecular pathological alterations underlying neurodegeneration invariantly pass through large-scale brain networks, as a class-wide phenomena affecting each neurodegenerative disease (Seeley et al., 2009). The impairment of large-scale brain networks represents the endpoint of a chain reaction, where the perturbation of molecular processes at the microscale level propagates through the mesoscale to eventually affect the macroscale level. At the microscale level, the abnormal protein assemblies, that are the very basis of the neurodegenerative process alter, for example, receptor expression, neurotransmitters release, and synaptic plasticity, producing synaptic dysfunction and failure (Bellucci et al., 2015). In the long term, synaptic impairment alters neuronal functioning by affecting activity-dependent signaling and gene expression, also producing distributed effects, at the mesoscale, on local neuronal circuits (Palop et al., 2006). Dysfunction in specific brain circuits eventually reverberates onto distant brain regions, resulting in disintegration of large-scale brain networks (Palop et al., 2006).

The idea of an “*active*” role for neuron-to-neuron interconnections in the spread of pathology stems from the observation that stereotypical patterns of pathology spreading are detectable in every neurodegenerative disease (see Brettschneider et al., 2015). Sequential stages of pathology spreading have been identified from post-mortem data, suggesting that propagation of pathology follows highly specific topographies (Braak and Braak, 1991; Braak et al., 2003, 2006; Brettschneider et al., 2013). Specifically, it was shown that tau spreads from the locus coeruleus to the transentorhinal cortex to cortical areas (Braak and Braak, 1991; Braak et al., 2006); amyloid plaques, from the neocortex to subcortical and brainstem regions (Braak and Braak, 1991); Lewy bodies (composed of immunoreactive α-synuclein), from the olfactory bulbar/dorsal motor nucleus of the vagus nerve through the basal forebrain to the neocortex (Braak et al., 2003); TAR DNA-binding protein 43 (TDP-43) pathology, from the agranular motor cortex to brainstem motor nuclei and spinal cord, eventually reaching the neocortex in later disease stages (Brettschneider et al., 2013). This *post-mortem* evidence is complemented by recent *in vivo* and *in vitro* studies, demonstrating that pathological proteins, similarly to prions, spread trans-synaptically along neuronal interconnections (Dujardin et al., 2014; Song et al., 2014; Narasimhan et al., 2017). Notably, injection of pathological proteins triggers protein spreading to spatially remote but anatomically connected brain regions, and the pattern of spreading depends only on the site of injection and on the neural connectome at that specific site of injection, and not on the type of protein strain (Narasimhan et al., 2017). Brain networks are thus active players in pathology spreading, setting the topographical constraints according to which pathology can propagate from its initial site of aggregation (Zhou et al., 2013). Available *in vivo* studies support this view, as spreading of both pathology and neurodegeneration map onto functional and structural brain networks (Seeley et al., 2009; Schmidt et al., 2016; Franzmeier et al., 2019). In this framework, brain connectomics is a powerful tool to investigate and predict the pattern of long-distance pathology spreading, as pathology spreading is strictly dependent on the topology of the underlying brain connectome (Fornito et al., 2015).

Although both the “*active*” and “*passive*” conceptualizations of brain networks are backed up by solid evidence, it stands to reason that their relevance might change along the time course of the disease. We can hypothesize that, at the very beginning of the neurodegenerative processes, the brain connectome would indeed act as an hard-wired “roadmap,” determining the pattern of pathology spreading (Zhou et al., 2013). Early on, pathological changes would affect connectome functional and structural properties, disrupting the “healthy” neuronal pathways and brain networks (Prescott et al., 2014). At this stage, pathology-related alterations of the brain connectome would progressively superimpose on the “original” connectome, dynamically interacting with the pre-morbid brain architecture, to determine subsequent spreading of the disease.

## Pet Molecular Connectivity

Molecular evidence on brain networks and connectivity pathways, obtained from PET imaging data, is now becoming increasingly available. The first PET connectivity studies, tracing back to the 1980s, took into account [18F]FDG–PET data to investigate *metabolic* molecular connectivity, defined as the association between inter-regional metabolic demands on the assumption that regions with similar metabolic demands are functionally associated (Horwitz et al., 1984). These pioneering studies remained “isolated experiments,” and were not replicated until the last decade, when the number of molecular metabolic connectivity studies steeply increased, in a “renaissance” (Yakushev et al., 2017) mainly driven by the renewed interest in the newborn field of connectomics. Together with [18F]FDG–PET metabolic connectivity studies, the application of connectivity approaches has now been extended to other PET targets, including neurotransmission systems. So far, molecular connectivity approaches have demonstrated novel network-level alterations in a wide range of neurodegenerative disorders. Crucially, the combination of connectivity approaches with PET molecular data provides extremely specific results on the underlying target, thus overcoming the “lack of specificity” typical of functional connectivity, as estimated from fMRI (Hahn et al., 2018). It is expected that molecular connectivity will greatly broaden the field of connectomics, providing an integrated, network-oriented, and biologically rooted, perspective, leading to a deeper understanding of the complexity of the brain architecture (Veronese et al., 2019).

### Basic Principles and Methods in PET Molecular Connectivity

**FIGURE 1:**
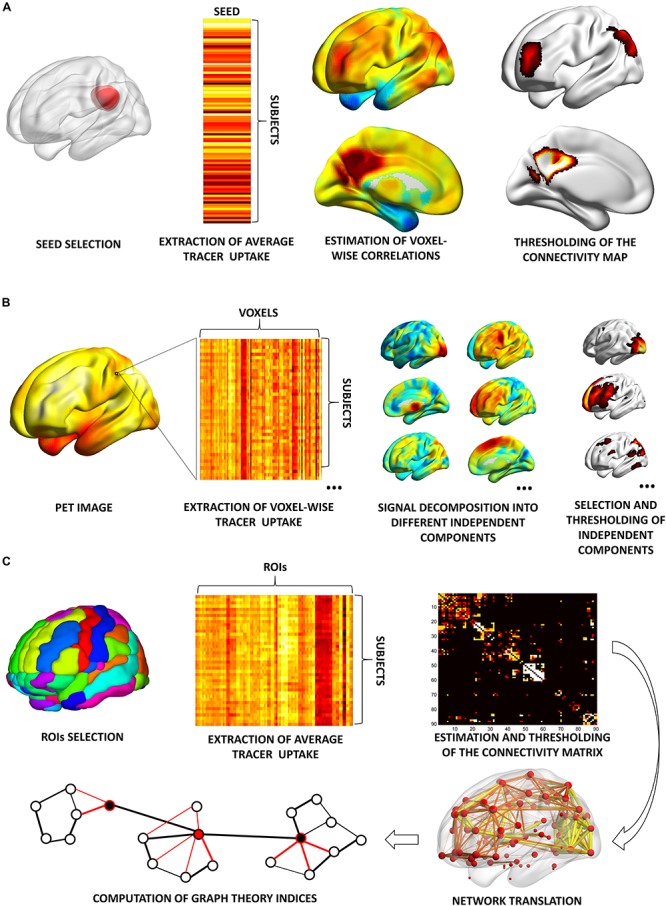
Schematic representation of the most common analytical approaches for molecular connectivity modeling. **(A)** Seed correlation analysis: molecular connectivity estimation is performed from a specific seed of interest, selected by the researcher. Here, the seed corresponds to a cluster encompassing the precuneus/posterior cingulate cortex; the resulting connectivity map corresponds to the default mode network. **(B)** Independent component analysis: whole-brain tracer uptake signal is decomposed into multiple statistically independent components, at voxel-wise level. The number of extracted components is set by the researcher. Here, *N* = 20 components are extracted (only six are shown for visualization purposes). By comparing the topography of the identified components with known anatomo-functional networks, the researcher can then select the components of interest. Here, three selected components are shown for visualization purposes, corresponding to the primary visual network, executive network, and default mode network. **(C)** Partial correlation analysis: molecular connectivity estimation is performed from ROIs selected by the researcher. Here, the ROIs comprehensively cover the whole brain. If the number of subject is smaller than the number of ROIs, sparse inverse covariance estimation (SICE) is used to estimate a connectivity matrix. This is then translated into a network, where nodes are represented by ROIs and edges by molecular connections. Here, a weighted connectivity matrix and weighted networks are shown, with edges computed at three different network densities. A wide array of graph theory metrics can then be estimated. BrainNet Viewer (http://www.nitrc.org/projects/bnv/) was used for rendering (Xia M. et al., 2013). ROIs, regions of interest.
(ii) *Independent component analysis* (*ICA*): this voxel-wise approach is based on the multivariate decomposition of PET signal across the brain (Di et al., 2012), under the assumption that PET signal can be described as a mixture of statistically independent components (Pagani et al., 2017). This approach allows identification of highly coherent brain networks in a data-driven fashion, without requiring any *a priori* selection of specific ROI (Figure 1B). Still, researcher’s intervention is needed to set the number of components to be extracted, and to select components with pathophysiological/anatomo-functional meaning, while discarding unimportant components of pure statistical noise. Although ICA represents the election method for connectivity analysis with fMRI data, contrasting results have emerged on its application to [18F]FDG–PET data for large-scale network estimation (Di et al., 2012; Savio et al., 2017).
(iii) *Partial correlation analysis:* this ROI-based approach allows to compute a comprehensive “connectivity matrix” following selection of a series of target regions, either based on a specific *a priori* hypothesis, i.e., ROIs belonging to a specific anatomo-functional system of interest or in a data-drive fashion, i.e., ROIs covering the whole brain (Figure 1C). This approach allows to estimate the degree of linear association between each couple of selected ROIs, after factoring out the contribution of all remaining ROIs to the target association. As such, partial correlation analysis overcomes the limitations of simple correlation analysis, that, by capturing pairwise information only, cannot characterize the effects of multiple brain regions interacting together (Huang et al., 2010). This method has subsequently been refined into a more advanced approach, known as sparse inverse covariance estimation (SICE) (Huang et al., 2010). The advantage of SICE is that it allows to estimate molecular connectivity even when the number of subjects included in the analysis is smaller than the number of ROIs, a relatively frequent scenario in PET studies (Huang et al., 2010). This is crucial particularly in connectivity studies for connectome assessment, where an elevated number of ROIs, covering the whole-brain, are selected. Once the whole-brain connectivity matrix is estimated through SICE, graph theory indices can be eventually computed, molecular hubs and modules identified, and changes in nodal and global network properties assessed [see Bullmore and Sporns (2009) for a review on graph theory indices].

Different analytical approaches have been implemented for molecular connectivity modeling (Yakushev et al., 2017). Three main analytical approaches are commonly used to estimate PET molecular connectivity:

(i) *Seed correlation or interregional correlation analysis* (*IRCA*): this voxel-wise approach relies on the selection of a region of interest (ROI), or seed, from which the average value of tracer uptake is extracted (Figure 1A). The correlation between average uptake in the seed and uptake in each voxel in the rest of the brain is then tested (Lee et al., 2008), to obtain an estimation of the connectivity profile, or connectivity map, of the seed of interest. This method yields a certain flexibility, as the researcher can select the ROI, or seed, in either a data-driven fashion (e.g., Morbelli et al., 2013; Iaccarino et al., 2018) or based on an *a priori* hypothesis (e.g., Ballarini et al., 2016; Malpetti et al., 2018). In the former case, the seed is derived directly from data analysis, usually by inputting, as seed, the cluster derived from a first round of univariate analyses (e.g., Morbelli et al., 2013; Iaccarino et al., 2018). In the latter case, this method has been commonly used to estimate large-scale brain networks in [18F]FDG–PET molecular connectivity studies (e.g., Ballarini et al., 2016; Malpetti et al., 2018). Resulting networks have similar topographies to the ones obtained with resting state-fMRI (Passow et al., 2015).

Although these approaches are methodologically quite different, they all rely on the assessment of regional co-variation in PET tracer uptake *across subjects* to estimate molecular connectivity (Figure 1). This is quite different from fMRI studies, where the availability of a time series for each subject allows to estimate functional connectivity based on regional co-variation of BOLD signal, through time, *within the same subject*. Since a times series is not available in PET studies, i.e., parametric PET images are inherently “static,” molecular connectivity studies always rely on the identification of patterns of inter-subject co-variation of regional tracer uptake. This will be further detailed in the last paragraph of the present review.

Independent of the analytical method used to estimate molecular connectivity, and the resulting outcome, i.e., seed-based maps of connectivity, mutually independent networks or connectivity matrices, results of molecular connectivity studies are usually translated into a common lexicon, with reference to *decreased* or *increased* connectivity. This is usually achieved through statistical comparison of connectivity metrics (e.g., network topography, connectivity stregth, and number of significant connections) between the target population and a reference group of healthy controls. At a basic level, interpretation of decreased and increased connectivity is quite straightforward, i.e., decreased (or increased) connectivity of a region to another indicates that the former region has a tracer uptake that is less (or more) associated with tracer uptake in the latter. At a more informative level, however, interpretation of connectivity changes becomes non-trivial and varies greatly depending on the type of PET tracer being used. In [18F]FDG–PET studies, similarly to fMRI studies, connectivity changes are usually interpreted in terms of function: connectivity decreases indicate functional disconnection between regions, while connectivity increases indicate increased functional coupling between regions. Interpreting the significance of connectivity increases is particularly non-trivial (Pievani et al., 2014): when increased connectivity affects metabolically preserved brain regions, it might be indicative of a “beneficial” compensatory process, with recruitment of brain regions that are still functional; when increased connectivity affects metabolically impaired brain regions, it might be indicative of a common underlying pathological process, conjunctly affecting metabolism of multiple brain regions in a similar fashion. For tracers targeting *pathology*, molecular connectivity increases can be expected with progressive pathology spreading. For tracers targeting *neurotransmission*, interpretation strictly depends on the specific neurotransmission system and target being studied: connectivity decreases might be indicative, for example, of selective denervation from the neurotransmitter nuclei projecting to the target regions being evaluated. Of note, changes in molecular connectivity can, but *do not* necessarily, reflect changes in anatomical connections between regions. Although anatomical disconnection between regions would likely result in changes in molecular connectivity, changes in molecular connectivity can be expected also without structural disconnection. Accordingly, and similarly to fMRI functional connectivity (Smith et al., 2013), molecular connections reflect both direct or indirect (polysynaptic) connections between regions, i.e., regions can be functionally connected, with or without a direct anatomical connection.

### Molecular Connectivity Studies Targeting Brain Metabolism

The investigation of brain metabolic connectivity has its foundation in the principle that regions whose metabolism is associated are functionally interconnected (Horwitz et al., 1984). This assumption stems from a pioneering study, demonstrating that metabolic connectivity results are largely consistent with known anatomo-functional data (Horwitz et al., 1984). Among the different declinations of molecular connectivity, metabolic connectivity theoretically represents the one most closely akin to functional connectivity, as assessed by fMRI. Available evidence suggests a close link between resting-state functional brain connectivity, as measured by resting-state fMRI, and glucose consumption, as measured by [18F]FDG–PET (Riedl et al., 2014; Passow et al., 2015). In a recent cross-modal study, it was shown that network properties of the brain metabolic covariance network indeed resemble that of the fMRI network, retaining the small-worldness property typical of functionally relevant organizations, as opposed to the randomness typical of structural organizations (Di et al., 2017). Direct comparison of the topography of brain networks as identified by [18F]FDG–PET vs. resting-state fMRI provided, however, contrasting results. Passow et al. (2015) demonstrated remarkably overlapping patterns of functional and metabolic connectivity seeding from the posterior cingulate gyrus, clearly corresponding to the topography of the default mode network (Passow et al., 2015). Savio et al. (2017), using simultaneously acquired [18F]FDG–PET and resting-state fMRI data, expanded these findings, reporting again substantial overlap within the major large-scale brain networks (Savio et al., 2017). While these results seem to point at a common underlying neural substrate for functional and metabolic connectivity (Passow et al., 2015; Savio et al., 2017), Di and colleagues (2012) reported mostly dissimilar findings, especially pertaining the default mode network (Di et al., 2012). It must be noted that, while both [18F]FDG–PET and fMRI signals represent proxies of synaptic function, they record extremely different aspects of neural activity. fMRI BOLD signal measures changes in the relative levels of oxy- and deoxy-hemoglobin, and is coupled to neural activity through the hemodynamic response, a neurovascular mechanism aimed at increasing blood flow to cover the energy demands of local brain activity (Bullmore and Sporns, 2009). BOLD signal is dependent on a combination of oxidative metabolism, blood flow, and blood volume (Gauthier and Fan, 2019), and is also affected by vessels size (Liu, 2013). fMRI is thus inherently more dependent of neuro-vascular coupling, and less directly linked to synaptic function, as compared to [18F]FDG–PET (cf. Yakushev et al., 2017). [18F]FDG–PET signal measures glucose consumption and is coupled to neuronal activity through a specific biochemical pathway, where excitatory glutamate release in the synaptic cleft elicits activation of the sodium/potassium pump, stimulating glucose consumption via aerobic glycolysis (Stoessl, 2017). Notably, energy consumption represents a proxy for *directional* signaling, as increases in local metabolism are indicative of increased afferent neuronal activity (cf. Riedl et al., 2016). Interestingly, this observation can be exploited to estimate effective brain connectivity with a completely data-driven approach, by combining undirected signaling pathways estimated from fMRI, with the information on directionality derived from [18F]FDG–PET data (Riedl et al., 2016).

In the following sections, we review the available metabolic connectivity findings in the main neurodegenerative disease spectra.

*Amyloidopathies*/*Tauopathies* – Among metabolic connectivity studies, Alzheimer’s disease represents definitely the nosographic entity that has received the greatest attention. The first study of metabolic connectivity in neurodegenerative conditions, in the 1980s, was indeed performed on a small sample of patients with Alzheimer’s disease dementia (Horwitz et al., 1987). Subsequent studies have consistently shown reduced metabolic connectivity seeding from the posterior cingulate gyrus/precuneus (Morbelli et al., 2012; Ballarini et al., 2016; Herholz et al., 2017), crucially representing the main hub of the default mode network. This finding was replicated in both late- and early onset Alzheimer’s disease, with involvement of additional brain networks in the latter group (Ballarini et al., 2016). It remains to be determined whether the pattern of metabolic connectivity alterations differs among atypical presentations of Alzheimer’s disease. To this regard, preliminary data suggest that, in atypical variants, specific alterations in metabolic connectivity might co-exist with the typical default mode network dysfunction (Herholz et al., 2017). Notably, cross-sectional studies suggest that metabolic connectivity deficits become increasingly more pronounced as disease progresses, with a gradual disintegration of the default mode network from prodromal to overt disease phases (Pagani et al., 2017). Interestingly, the majority of these studies reported reduced metabolic connectivity in both hypo-metabolic and metabolically preserved brain regions, at consistence with the view that connectivity alterations can exceed local metabolic deficits (Clark and Stoessl, 1986). This is also in accordance with recent pathophysiological models of neurodegenerative diseases, suggesting that connectivity alterations can spread at long-distance, affecting brain regions that would be otherwise spared by pathology (Warren et al., 2013). Together with decreases metabolic connectivity, *increased* network connectivity has also been reported in Alzheimer’s disease, in association to reserve proxies such as education (Morbelli et al., 2013; Malpetti et al., 2017) and bilingualism (Perani et al., 2017). It has been suggested that these results might be indicative of a common compensatory mechanism, according to which lifelong protective factors promote stronger integration of large-scale brain networks, in spite of more severe hypometabolism (Yakushev et al., 2017). Of note, in a recent report in Alzheimer’s disease, we found that –in females only – high body mass index levels determine *decreased* connectivity in the very same brain networks involved in the above-mentioned compensatory mechanisms (Malpetti et al., 2018), suggesting a multi-factorial modulation of brain connectivity in Alzheimer’s disease.

As for primary tauopathies, to the best of our knowledge, only one metabolic connectivity study is available. In this study, Titov et al. (2017) reported that pathological metabolic connections tend to cluster in the frontal and temporal lobes of subjects with the behavioral variant of frontotemporal dementia (Titov et al., 2017).

Although patterns of focal metabolic alterations have been relatively well-characterized in the other primary tauopathies, namely corticobasal degeneration and progressive supranuclear palsy (e.g., Niethammer et al., 2014; Caminiti et al., 2017a), a network-level characterization of these diseases is still lacking.

**FIGURE 2:**
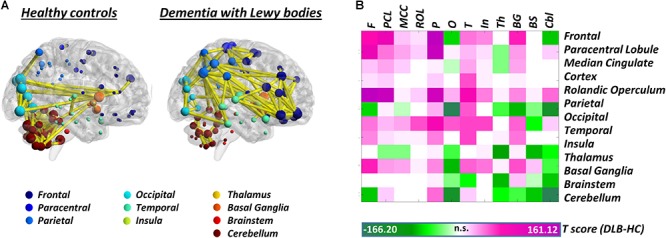
Whole-brain metabolic connectome in Dementia with Lewy bodies. **(A)** Brain connectivity graphs in healthy controls and patients with dementia with Lewy bodies. A global connectivity reconfiguration is evident in patients with dementia with Lewy bodies, with metabolic connectivity decreases mainly affecting occipital cortex, thalamus, and cerebellum. Only the strongest connections (density ≈ 3%) are shown (yellow edges). The size of each node depends on the node total number of connections, whereas the color indicates its anatomical localization. **(B)** The *T*-score matrix reports *T*-test statistics, derived from the direct comparison of the number of metabolic connections (within and between each macroarea) between patients and healthy controls, following a bootstrapping procedure. Connectivity decreases are indicated by negative *T*-scores (green); connectivity increases by positive *T*-scores (violet). BrainNet Viewer (http://www.nitrc.org/projects/bnv/) was used for rendering (Xia M. et al., 2013). Modified from Caminiti et al. (2017c). F, frontal; PCL, paracentral lobule; MCC, median cingulate cortex; ROL, rolandic operculum; P, parietal; O, occipital; T, temporal; In, insula; Th, thalamus; BG, basal ganglia; BS, brainstem; Cbl, cerebellum.

*Synucleinopathies* – In the spectrum of synucleinopathies, metabolic connectivity studies have focused mainly on Parkinson’s disease. Spetsieris et al. (2015) reported a perturbation of metabolic connectivity in the default mode network in late Parkinson’s disease, associated with subsequent development of cognitive dysfunction (Spetsieris et al., 2015). In a more recent study, we replicated these findings, and additionally showed that connectivity alterations, in Parkinson’s disease, go well beyond the default mode network, with perturbations of frontal connectivity in virtually each large-scale brain network (Sala et al., 2017). In the same study, we also assessed whole-brain connectome alterations, reporting connectivity decreases, locally and at long-distance, in the frontolateral cortex, opposed to connectivity increases, of possible compensatory significance, in occipital regions. This pattern of connectivity impairment was antithetical to the one observed in dementia with Lewy bodies, characterized by local and long-distance occipital connectivity decreases, and frontal connectivity increases (Caminiti et al., 2017c; Figure 2). Still, the two synucleinopathies showed a common backbone of connectivity alterations, involving cerebellum and mesencephalic–pontine regions, notably representing very early sites of α-synuclein aggregation into Lewy bodies, as the pathological hallmark of both diseases (Caminiti et al., 2017c; Sala et al., 2017). To the best of our knowledge, no metabolic connectivity studies are available in multiple system atrophy.

*Other* – Little is known on network-level molecular alterations in neurodegenerative diseases underlid by other pathological proteins, such as TDP-43 and huntingtin proteins. To this regard, the only available study using IRCA in the TDP-43 spectrum, specifically in amyotrophic lateral sclerosis, reported a positive association between metabolism in the midbrain and white matter in the corticospinal tract (Pagani et al., 2014). Still, results deriving from *group-level* metabolic connectivity analysis in amyotrophic lateral sclerosis should be interpreted with caution, as they might be affected by the intrinsic metabolic heterogeneity that characterizes this condition (Matías-Guiu et al., 2016; Sala et al., 2019). Further reflection on group inhomogeneity and molecular connectivity is reported in the last section of this review.

### Molecular Connectivity Studies Targeting Brain Neurotransmission Systems

Positron emission tomography studies targeting brain neurotransmission systems make up a significant proportion of molecular connectivity evidence. So far, the majority of these studies have focused on assessing network-level properties of neurotransmission systems in healthy controls and their alterations in neuropsychiatric disorders, with only a few studies available in neurodegenerative conditions. These studies are based on the assumption that cell firing and neurotransmitter release from a given neurotransmitter nucleus would affect the expression of tracer binding sites in the nucleus’ target regions (Hahn et al., 2018), and would do so in a similar way for targets innervated by the same nucleus. The first attempt to apply multivariate methods to tracers for neurotransmission was carried out by Cervenka et al. (2010), assessing patterns of correlations between striatal and extrastriatal dopaminergic D2-receptor in 16 control subjects. They found that the pattern of correlations of the radioligands [11C]raclopride and [11C]FLB457 was consistent with the known biochemical architecture of the dopaminergic systems, reflecting the segregation between nigrostriatal, mesolimbic, and mesocortical pathways (Cervenka et al., 2010). Additional studies followed, extending the investigation of molecular connectivity to the serotoninergic (e.g., Hahn et al., 2014; Tuominen et al., 2014; Pillai et al., 2018) and μ-opioid systems (Tuominen et al., 2014). During the last years, these studies have progressively moved from “simple” seed-based correlative approaches, to the implementation of methods borrowed from functional/structural connectomics, adopting graph theory measures to estimate neurotransmission systems’ organization properties. From the theoretical standpoint, these studies have progressively situated their results in the framework of connectomics, enriching “system level” knowledge with “molecular-level” information (see Tuominen et al., 2014). Altogether, this evidence shows that PET can reliably reconstruct brain connectivity patterns within and between neurotransmitter systems, providing *in vivo* access to the biochemical architecture of the brain.

To the best of our knowledge, only one study has measured molecular connectivity alterations of neurotransmission systems in neurodegenerative diseases. In this study, Caminiti et al. (2017b) investigated the nigrostriatal and mesolimbic dopaminergic pathways in a series of Parkinson’s disease patients, reporting a severe reduction in dopaminergic connectivity between substantia nigra and putamen, a portion of the striatum known to be early affected by nigral denervation (cf. Caminiti et al., 2017b). The mesolimbic network was also affected, with loss of connectivity between homotopic regions, but no change in connectivity between the ventrotegmental area and its subcortical targets. These results support the view of Parkinson’s disease as a disconnection syndrome, with axonal damage representing an early occurrence in the degeneration of the nigrostriatal system (Caminiti et al., 2017b; Fazio et al., 2018). Other studies, based, however, on single photon emission computed tomography (SPECT) radioligands for dopaminergic transporter imaging also adopted a similar approach (Premi et al., 2016, 2017). These studies showed abnormal patterns of subcortico-cortical molecular connectivity in Parkinson’s disease (Premi et al., 2016, 2017), with additional putaminal–cingulate disconnection specifically associated with the presence of impulse control disorder (Premi et al., 2016).

**FIGURE 3:**
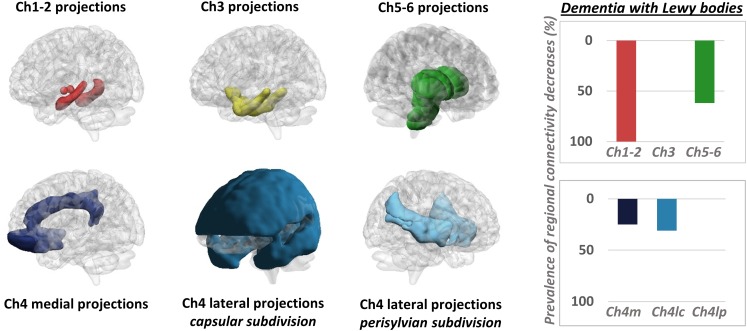
Metabolic connectivity targeting cholinergic pathways. Left panel: selection of ROIs for assessment of metabolic connectivity within the major cholinergic pathways. The first row shows regions supplied by basal forebrain (Ch1-2; Ch3) and brainstem (Ch5-6) nuclei. The second row shows the three cholinergic pathways projecting from the nucleus basalis of Meynert (Ch4). Right panel: histograms show the prevalence of regional connectivity decreases in dementia with Lewy bodies, in each cholinergic pathway. Prevalence of metabolic connectivity decreases in each pathway was computed as the number of regions, within the pathway, presenting with significantly decreased metabolic connectivity, divided by the total number of regions belonging to that pathway. Only connections within each pathway (i.e., between regions innervated by the same cholinergic nucleus) were taken into account to compute the prevalence of connectivity decreases *within* each pathway. Prevalence of metabolic connectivity decreases was higher in regions supplied by Ch1–Ch2 nuclei (100% of regions presenting with significantly decreased metabolic connectivity within this pathway) and Ch5–Ch6 nuclei, with additional involvement of the medial and lateral projection (capsular subdivision) of the nucleus basalis of Meynert (Ch4). BrainNet Viewer (http://www.nitrc.org/projects/bnv/) was used for rendering (Xia M. et al., 2013). Modified from Caminiti et al. (2017c). Ch4m, Ch4 medial projections; Ch4lc, Ch4 lateral projections, capsular subdivision; Ch4lp, Ch4 lateral projections, perisylvian subdivision.

It must be mentioned that a few PET studies adopted an alternative strategy, mapping changes in neurotransmission pathways via estimation of molecular *metabolic* connectivity in selected brain regions (see Figure 3 for an example). This approach is based on the assumption that energy consumption is influenced by multiple pathological events, notably including altered neurotransmission (Perani, 2014) and builds on the evidence of a significant coupling between neurotransmission impairment and integrity of metabolic networks (Holtbernd et al., 2015). Using this approach, it was shown that metabolic connectivity alterations can be detected within the nigrostriatal dopaminergic and mesocorticolimbic system, in both Parkinson’s disease (Sala et al., 2017) and dementia with Lewy bodies (Caminiti et al., 2017c), with reconfigurations more prominent in the latter. Using a similar rationale, Verger et al. (2018) reported a disruption of metabolic connectivity in regions belonging to the mesocorticolimbic system, specifically in Parkinson’s disease patients presenting with impulse control disorder (Verger et al., 2018). Further application of this method extends to the cholinergic pathways, where derangement was reported running along cholinergic projections from the basal forebrain and brainstem nuclei, in dementia with Lewy bodies (Caminiti et al., 2017c; Figure 3).

### Molecular Connectivity Studies Targeting Brain Pathology

The most recent application of brain molecular connectivity involves the use of PET tracers for brain amyloidosis and tau pathology. Contrarily to molecular connectivity studies targeting metabolism and neurotransmission systems, where the focus is on assessing the *effects* of pathology on the energetic and biochemical architecture of the brain, this branch of molecular connectivity clearly builds upon the hypothesis of brain networks as *active players* in the spreading of pathology (see above, paragraph: “The brain as a network”). Since pathology spreading maps onto the underlying structural brain networks, superimposing on the brain connectome, pathology spreading might show, *per se*, “network” properties, and thus be modeled with network-level approaches. Accordingly, recent studies have started to make reference to the “amyloid network” and “tau network” (Sepulcre et al., 2013; Hoenig et al., 2018). Quite a few molecular connectivity studies using established PET tracers for amyloidosis are available, while molecular connectivity studies with tau tracers are emerging.

As a side note, it must be underlined that although the models of connectivity-based pathology spreading hold true for tau pathology and amyloid pathology in the form of oligomers (Ahmed et al., 2014; Domert et al., 2014), some evidence suggests that spreading of extracellular fibrillary amyloid plaques, targeted by currently available PET tracers, might be led by spatial proximity and not by brain connectivity (Mezias and Raj, 2017). Still, this finding remains controversial.

*Amyloidosis* – In the first study targeting the “amyloid network,” Sepulcre et al. (2013) demonstrated that it is possible to use molecular connectivity data to derive information on putative mechanisms of amyloid spreading (Sepulcre et al., 2013). They showed that “hubs” in the amyloid network act as seeding nodes from where amyloid accumulation spread to more peripheral regions (Sepulcre et al., 2013). The hubs that they identified, including the medial temporal lobe and orbitofrontal cortex, are partially consistent with the early regions of amyloid accumulation, as defined by post-mortem studies (Braak and Braak, 1991; Thal et al., 2002). Braak and Braak (1991) described the presence of amyloid pathology in basal portions of the isocortex (including orbitofrontal cortex), together with mild involvement of medial temporal lobe structures, already in Stage A (Braak and Braak, 1991). Thal et al. (2002) reported involvement of the neocortex first (including orbitofrontal cortex) (Phase 1), followed by medial temporal lobe structures already in Phase 2 (Thal et al., 2002). Although presence of amyloid pathology in the medial temporal lobe is described as an early (Stage A according to Braak’s staging) or relatively early (Phase 2 according to Thal’s phases) event, it is worth noting that medial temporal amyloid burden remains mild even in the latest stages of amyloid accumulation (Braak and Braak, 1991). Due to the mismatch between timing and severity of amyloid pathology, univariate approaches based on severity of amyloid pathology only would necessarily *not* be able to identify the same amyloid hubs as those reported by Sepulcre et al. (2013) using multivariate connectivity techniques. Interestingly, a recent study has shown that the localization of pathological hubs in the amyloid network might not be invariant, but differ across different variant of the Alzheimer’s disease spectrum (Leyton et al., 2015). This result contrast with the well-established homogeneity of amyloid burden reported across different conditions (Iaccarino et al., 2017a). Further studies on large population of patients are necessary to better define these aspects.

Other recent studies have compared nodal and global properties of the amyloid network in healthy controls, subjects with mild cognitive impairment and patients with Alzheimer’s disease dementia (Jiang et al., 2015; Son et al., 2015; Duan et al., 2017). These studies have however reported contrasting results, with respect to both the direction and the localization of the amyloid network’s alterations, possibly due to differences in the proportion of amyloid positive/negative cases in the tested cohorts (Duan et al., 2017). To this regard, a recent study adopted a radically different approach, classifying subjects not on the basis of their clinical status, but on a multi-modal biomarker-based amyloid staging, allowing classification of subjects into “negative,” “early,” and “late” amyloid accumulators (Pereira et al., 2017). This elegant study has shown that the amyloid network is characterized by a “community” of strongly interconnected regions, notably partly overlapping with the default mode network, invariantly detectable across all amyloid stages (Pereira et al., 2017). Interestingly, as amyloid accumulation becomes more severe, this community progressively expands to include additional neocortical regions (Pereira et al., 2017), suggesting that changes in network topology reflect amyloid pathology progression and spreading.

*Tau pathology* – To the best of our knowledge, only one study has investigated molecular connectivity using tau tracers. In this extremely recent study, Franzmeier et al. (2019) reported that tau covariance is linearly associated with functional connectivity, independently of spatial proximity (Franzmeier et al., 2019). These results provide *in vivo* support to the view that patterns of tau spreading map onto the underlying connectome, and that tau spread preferentially across connected (but not necessarily adjacent) brain regions (Franzmeier et al., 2019). Notably, these results were confirmed not only in Alzheimer’s disease, but also in normal aging and cerebrovascular cognitive impairment, suggesting a strong coupling between tau propagation and functional connectivity, independent of amyloidosis (Franzmeier et al., 2019). Still, these findings need to be interpreted with caution, as significant off-target binding has been reported for the [18F]AV–1451 tau tracer used in this study (cf. Lemoine et al., 2018).

*α-Synuclein pathology* – Currently, there is no validated tracer to *in vivo* measure α-synuclein pathology. Several compounds have been investigated, but subsequently discarded as they did not show sufficient *in vivo* binding or acceptable selectivity for the target (Jovalekic et al., 2017; Mathis et al., 2017). To remedy the lack of information on molecular connectivity alterations within the α-synuclein network, we used an alternative approach based on [18F]FDG–PET, following the same rationale explained above. We mapped [18F]FDG–PET *metabolic* connectivity in a series of *a priori* selected brain regions, as derived from currently available neuropathological models of α-synuclein staging (Braak et al., 2003). Using this approach, we showed that metabolic connectivity alterations map onto the underlying pathology, with the most severe alterations involving regions early affected by α-synuclein pathology, in both Parkinson’s disease (Sala et al., 2017) and dementia with Lewy bodies (Caminiti et al., 2017c). In spite of the relative lack of specificity of the [18F]FDG–PET signal, these results show that α-synuclein pathology leaves a unique imprint on metabolic connectivity.

### Limitations and Future Directions in Molecular Connectivity – Getting at the Single-Subject Level?

Although compelling, the net majority of molecular connectivity results are based on group-level analyses, due to the inherently “static” nature of PET images – containing either tracer uptake values averaged over a certain time window or parametric values derived from the tracer’s dynamics. As a consequence, quantified PET images do not possess the temporal “dynamic” component that is typical, for example, of fMRI data (Yakushev et al., 2017). This limitation makes a within-subject “fMRI-like” analysis of PET images impossible. For this reason, molecular connectivity analysis is necessarily performed at group level. It follows that the reliability of molecular connectivity findings depends on the definition of a relatively homogeneous cohort, on the one hand, and on the normalization of between-subject differences, e.g., due to variability in image acquisitions, on the other hand (cf. Veronese et al., 2019). Currently, little is known on the effect of sample heterogeneity on molecular connectivity results, and, more in general, on test–retest reliability and reproducibility of molecular connectivity findings. Encouragingly, a very recent validation study has shown good reproducibility of molecular connectivity results, obtained with an ROI-based correlative approach and graph theory, and confirmed using three different tracers, suggesting a general applicability within typical experimental settings (Veronese et al., 2019). More studies are urgently needed to confirm and extend these findings using other analytical methods and other tracers, and address the effect of other experimental variables, e.g., sample size, on molecular connectivity results.

Together with validation studies, derivation of individual metrics to quantify molecular connectivity alterations at the single-subject level represents a top priority in the field of neurodegenerative diseases. Availability of single-subject metrics would allow to test the value of molecular connectivity as a biomarker of diagnostic and prognostic interest, and to perform correlative analysis, e.g., to investigate the association between individual connectivity metrics and other imaging parameters, clinical symptoms/neuropsychological deficits or, eventually, as complementary outcome measures to evaluate the effects of new emerging treatments (e.g., Fortier et al., 2019). Although the estimation of molecular connectivity patterns necessarily requires – as a first step – a group-level analysis, different approaches have been developed to subsequently derive information at the single-subject level. So far, it has been shown that single-subjects connectivity metrics can be estimated following group-level molecular connectivity analysis, from: (i) independent connectivity analysis, followed by computation of the loading coefficient (Savio et al., 2017); (ii) partial correlation analysis, followed by a bootstrapping procedure that allows to obtain a distribution of connectivity parameters, still at group-level, but that can nevertheless be used for correlative analysis (Franzmeier et al., 2019); and (iii) SICE, using the equation of the multivariate Gaussian distribution (Titov et al., 2017). Finally, a radically different approach has been proposed, involving the use of dynamic PET data, traditionally used only to estimate the final parametric “static” PET image (Passow et al., 2015; Tomasi et al., 2017). This approach takes full advantage of the dynamic nature of PET data, using the temporal fluctuations in tracer uptake to estimate within-subject “temporal” molecular connectivity, adopting an analytical pipeline similar to the one used in resting-state fMRI analysis (Passow et al., 2015; Tomasi et al., 2017). This allows to obtain a “direct” estimation of the patterns of connectivity alterations at single-subject level, similarly to fMRI data. Still, it has been noted that results obtained with this approach, based on estimation of molecular connectivity using tracer *dynamics*, might lack biological specificity (Veronese et al., 2019), in particular for [18F]FDG tracer. In general, tracer dynamics are dependent not only on tracer’s specific binding, but also on its non-specific binding and its delivery properties (Veronese et al., 2019), making the interpretation of results obtained with such approach less straightforward, as compared to “traditional” approaches based on parametric *static* PET images. In addition, the use of individual frames of PET acquisitions might harbor other limitations; most importantly, the necessarily shorter duration of individual frames would yield lower signal-to-noise ratio compared to static images derived from the whole acquisition time.

## Conclusion

Molecular connectivity represents a powerful tool to investigate the pathophysiology of neurodegenerative diseases, providing *in vivo* access to a potentially endless series of biological processes, from cellular metabolism, to neurotransmission, to aggregation of pathological proteins. The potential of molecular connectivity is still emerging, and might possibly be endless, as new areas of application would arise in parallel with the validation of new tracers for new biological targets. A recent development is related to the possibility to assess not only brain glucose metabolism, as measured by [18F]FDG–PET, but also brain ketone metabolism, using [11C]AcAc–PET (Courchesne-Loyer et al., 2017). Although contribution of ketones to brain energy requirements is scarce under standard conditions (<5%), recent evidence suggests that ketone metabolism might hold relevance in both aging and Alzheimer’s disease (Croteau et al., 2018). Combining [18F]FDG and [11C]AcAc tracers would allow to test for similarities and differences between glucose-based and ketone-based brain metabolic networks. Another interesting development would concern the possibility to assess molecular connectivity using tracers for synaptic activity, such as the newly developed [11C]UCB-J tracer (Finnema et al., 2016). This would give us a direct access to molecular networks of “pure” neural activity, without the mediation of BOLD signal or metabolic processes (Heurling et al., 2017).

Thus, the progressive implementation of molecular connectivity techniques, with possibly unlimited applications brought by the development of new PET tracers, will allow unique breakthroughs in our understanding of neurodegenerative mechanisms. Once approaches to estimate single-subject brain molecular connectivity will become well-established, brain connectivity signatures might hold promises to be validated as biomarkers for diagnostic and prognostic use, or, eventually, as complementary outcome measures to evaluate the effects of new emerging treatments. Hopefully, molecular connectivity studies “will gain momentum, and deservedly so!” (Yakushev et al., 2017).

## Author Contributions

AS performed the PubMed search and wrote the review. DP wrote the review and critically revised the article for intellectual content.

## Conflict of Interest Statement

The authors declare that the research was conducted in the absence of any commercial or financial relationships that could be construed as a potential conflict of interest.
